# Idiopathic linear leukoplakia of gingiva: A rare case report

**DOI:** 10.4103/0972-124X.75918

**Published:** 2010

**Authors:** N. Sapna, K. L. Vandana

**Affiliations:** *Department of Periodontics, D.A.P.M R.V. Dental College, Bangalore, India*; 1 *Department of Periodontics, College of Dental Sciences, Davangere, Karnataka, India*

**Keywords:** Gingiva, idiopathic, linear leukoplakia, non-malignant

## Abstract

White lesions of the oral cavity are not uncommon though majority of them are benign. This case report documents a rare case of idiopathic linear leukoplakia of gingiva with no apparent etiology. Initial examination revealed a non-scrapable linear white lesion on the marginal and papillary gingiva of upper right teeth region. Incisional biopsy was taken for pathologic evaluation. Patient was treated with routine oral hygiene procedures and excision of the lesions. The histopathological results demonstrated hyperparakeratinized/orthokeratinized hyperplastic oral epithelium with orthokeratin-filled clefts and with no dysplasia. Clinical results demonstrated no recurrence after electrosurgical intervention. This paper reports a rare case of idiopathic linear leukoplakia of gingiva which was non-dysplastic in nature. Electrosurgical treatment proved to be successful compared to surgical technique as there was no recurrence even after two years of follow-up.

## INTRODUCTION

Any condition that increases the thickness of the epithelium causes it to appear white by increasing the distance to the vascular bed. Most often lesions appear white because of a thickening of the keratin layer, or hyperkeratosis. Other common causes of a white appearance include acanthosis, an increase in the amount of edema fluid in the epithelium, and reduced vascularity in the underlying lamina propria. Surface ulcerations covered by a fibrin cap can also appear white, as would collapsed bullae.[1]

The development of oral white patches is not uncommon, but, fortunately, the majority of lesions are due to benign conditions. However, a small percentage of white patches may represent either oral cancer or have an association with the likelihood of the development of oral cancer. The presence of sinister lesions cannot be assessed by clinical appearance alone and clinical diagnosis of any persistent white patch should therefore be confirmed histologically.[2]

Oral leukoplakia can be defined as “a predominantly white lesion of the oral mucosa that cannot be characterized as any other definable lesion; some oral leukoplakia will transform into cancer”.[3]

Leukoplakia can be subdivided according to etiology and clinical factors and one of the types is idiopathic leukoplakia where no etiology for the patch can be found.[4]

This paper describes a case of idiopathic linear leukoplakia involving the marginal and papillary gingiva with no apparent etiology.

## CASE DISCRIPTION AND RESULTS

### Clinical findings

A 40-year-old male patient came to the Department of Periodontics, College of Dental Sciences, Davangere, India, with a chief complaint of thin white lesion on the gums in the upper front region since 4 months. He noticed the lesion when he got his upper front teeth removed, which was placed irregularly on the inner side. The lesion was totally asymptomatic except for the esthetic concern of the patient. His medical history disclosed that he was a well-controlled hypertensive patient since four years. Patient did not smoke tobacco, or chew areca nut. No history of trauma or surgery was disclosed in that particular region. However, patient gave history of vigorous brushing and taking hot beverages.

**Figure 1:**
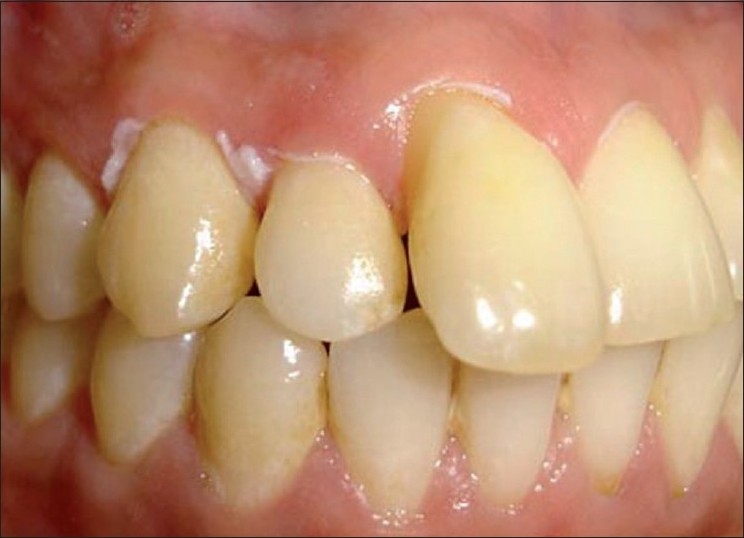
First visit, buccal linear white lesions of marginal gingival in relation to 11, 12, 13

**Figure 2:**
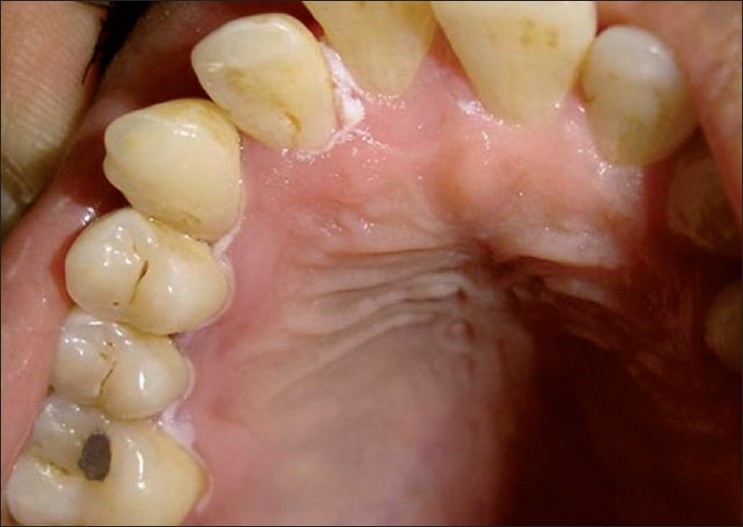
First visit, palatal linear white lesions in relation to 11, 12, 13, 15 and 16

Clinical examination revealed a linear white lesion on the upper right region involving marginal and papillary gingiva of both labial and palatal side. It was more prominent on labial aspect of 11, 12, 13 and 16 and palatal aspect of 11, 12, 13, 15 and 16 [Figures 1 and 2]. Lesions were non-scrapable. The patient’s oral hygiene was good. Radiographic examination showed normal findings. Laboratory investigations revealed hemoglobin level of 13.4 g%, a white blood cell count of 5200 cells/mm^3^ and normal platelet count. Random blood sugar was within normal limits [Table 1]. There was no evidence of skin lesions. HIV testing showed negative results.

**Table 1:** Complete hemogram

	Report	Normal values
Hb estimation	13.4 g%	14–18 g% (males 11.5–6.5 mg% (females)
ESR count	30 mm/m	0–15 mm (males) 0–20 mm/1 h (females)
RBC count	5.1 millus mm^3^	4.5×10^5^–6×10^5^/mm^3^
Platelet count	2.0 lacs/mm^3^	1×10^5^–4×10^5^/mm^3^
Total WBC count	5200 cells/mm^3^	4000–10,000/mm^3^
Bleeding time	1 min 45 s	1–5 min
Clotting time	3 min 15 s	4–9 min
Neutrophils	58%	40–75%
Lymphocytes	36%	20–45%
Eosinophils	04%	01–04%
Monocytes	02%	02–08%
Basophils	00%	00–01%
Blood sugar		
Random blood sugar	126.0 mg%	70 – 160 mg%

### Histopathological findings

**Figure 3:**
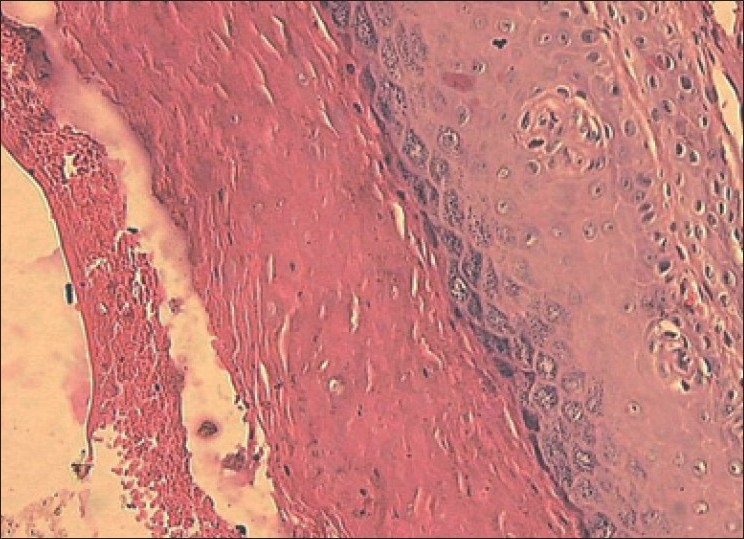
H and E stain of tissue biopsy during first visit showing hyperplastic oral epithelium, lymphocytic infiltration in connective tissue with no evidence of dysplasia

An approval from the institution’s review committee and an informed consent from the patient were obtained. An incisional biopsy of the lesion was performed in relation to labial gingiva of 13 and 16 and palatal gingiva of 11 and 14 with patient under local anesthesia. It was stained with hematoxylin and eosin and observed under light microscope. Epithelium showed exophytic proliferations lined by hyperparakeratinized / orthokeratinized hyperplastic oral epithelium with few broad and deep clefts filled with orthokeratin. Underlying connective tissue showed lymphocytic infiltration. There was no evidence of dysplasia [Figure 3].

By correlating clinical and histopathological findings the lesion was diagnosed as idiopathic linear leukoplakia of the gingiva.

### Treatment and follow-ups

**Figure 4:**
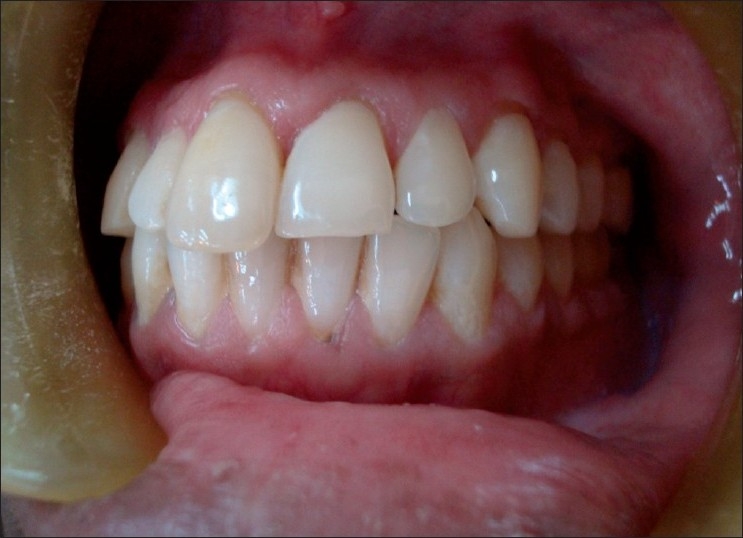
Recall visit, no recurrence of white lesion after two years of follow-up

The case was followed-up regularly for two years from August 2005 to August 2007. Scaling and oral hygiene procedures were performed regularly. The linear white lesions were excised using B.P. blade No. 15 under local anesthesia on the labial aspect. Palatal lesions were not excised this time, so that they could serve as reference lesions, if the labial lesions recurred similar to previous lesion. At one-month reevaluation, the labial linear white lesions had recurred. Once again the surgical excision was done on both labial and palatal aspect and sent for histopathological evaluation second time. Following the second surgical intervention, linear white lesion had recurred again at one month’s recall. Considering the patient’s esthetic concern and nondysplastic tissue changes in both the histopathological report, an attempt was made to eliminate linear white lesions using electrosurgery approximately 6 months after the first excision. The patient’s compliance was highly appreciable during the maintenance phase. The regular checkup over two years period did not reveal any recurrence of the white linear lesion and patient was satisfied with the treatment and the results [Figure 4].

## DISCUSSION

Oral white lesions reflect many different diseases and pathological changes. Some of them relate to diseases such as lichen planus and lupus erythematosus. Others are local changes with a clearly defined or highly probable etiology.

Leukoplakia is the most prevalent precancerous lesion of the oral mucosa. The label “leukoplakia” was coined by Schwimmer.[5] Approximately 70% oral leukoplakias are found on the lip vermillion, buccal mucosa and gingiva.[6]

Leukoplakia of the gingiva varies in appearance from a grayish white, flattened, scaly lesion to a thick, irregularly shaped keratinous plaque.[7] The cause of leukoplakia remains unknown though it can be associated with the use of tobacco. The lesions where the etiology is not apparent can be termed as idiopathic leukoplakia.[4]

In the present case, since histopathological reports did not show any dysplastic changes, premalignant and malignant lesions were not taken into considerations for the diagnosis.

Among reactive and inflammatory white lesions, frictional (traumatic) keratosis was not considered for the diagnosis since an identifiable source of mechanical irritation like rough or maladjusted dentures, sharp cusps and edges of broken teeth was not found. Though patient gave a history of hard brushing and consuming hot beverages, lesions did not resolve even after patient left these habits ruling out these habits as source for the lesion.

Chemical injuries were ruled out since patient did not give any history of usage of chemicals. Since patient did not smoke or chew tobacco, tobacco-induced keratosis was excluded from the diagnosis.

Infectious lesions like candidiasis, bacterial infections or viral infections were also ruled out since patient’s history was non-suggestive and also biopsy report did not show any changes.

The use of vigorous brushing by the patient would not lead to occurrence of lesions on the upper right side excluding the other quadrants especially the lower right quadrant. Considering the patient’s history, clinical and histopathological examination, the lesion can be termed as idiopathic linear leukoplakia of the gingiva.

Patient has been kept under observation and till date has not shown any recurrence of the lesion.

## CONCLUSION

This case report was considered, as idiopathic leukoplakia on the gingiva is a very rare finding. It was considered as idiopathic lesion as no etiology could be related to the present lesion. Further observations should be done to detect any recurrence of the lesion.

## References

[1] Bhattacharya I, Cohen DM, Silverman S (2003). Red and white lesions of the oral mucosa. Burket’s oral medicine diagnosis and treatment.

[2] Lamey PJ (1990). Oral medicine in practice: White patches. Br Dent J.

[3] Axell T (1996). Oral white lesions with special reference to precancerous and tobacco related lesions: Conclusion of an international symposium held in Uppsala, Sweden in 1994. J Oral Pathol Med.

[4] Axell T (1987). Occurrence of leukoplakia and some other oral white lesions among 20333 adult Swedish people. Community Dent Oral Epidemiol.

[5] Schwimmer E (1877). Some rare clinical pictures of oral and lingual mucosa. Arch Dermat Syph.

[6] Neville BW, Damm DD, Allen CM, Bouquot JE (2004). Epithelial pathology. Oral Pathology and Maxillofacial Pathology.

[7] Carranza FA, Hogan EL (2003). Gingival enlargement. Carranza’s clinical periodontology.

